# Supplementary material for: Migrating Myofibroblastic Iliotibial Band-Derived Fibroblasts Represent a Promising Cell Source for Ligament Reconstruction
Source: Int J Mol Sci. 2019 Apr 22;20(8):1972. doi: 10.3390/ijms20081972 (PMC6514966; doi:10.3390/ijms20081972)
Supplement: Supplementary file 1 [file ijms-20-01972-s001.pdf]

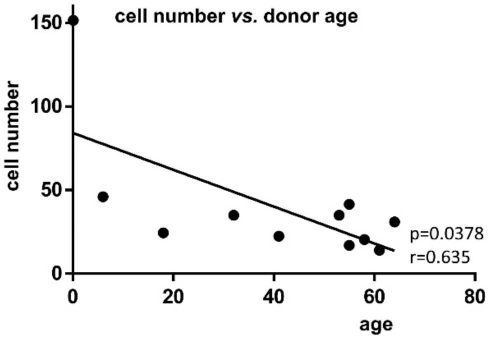

Supplementary Figure 1. Cell numbers versus the donor age in ITB tissue. There was a significant correlation. However, when excluding the very young donor (5 weeks) the interrelation was no longer significant.

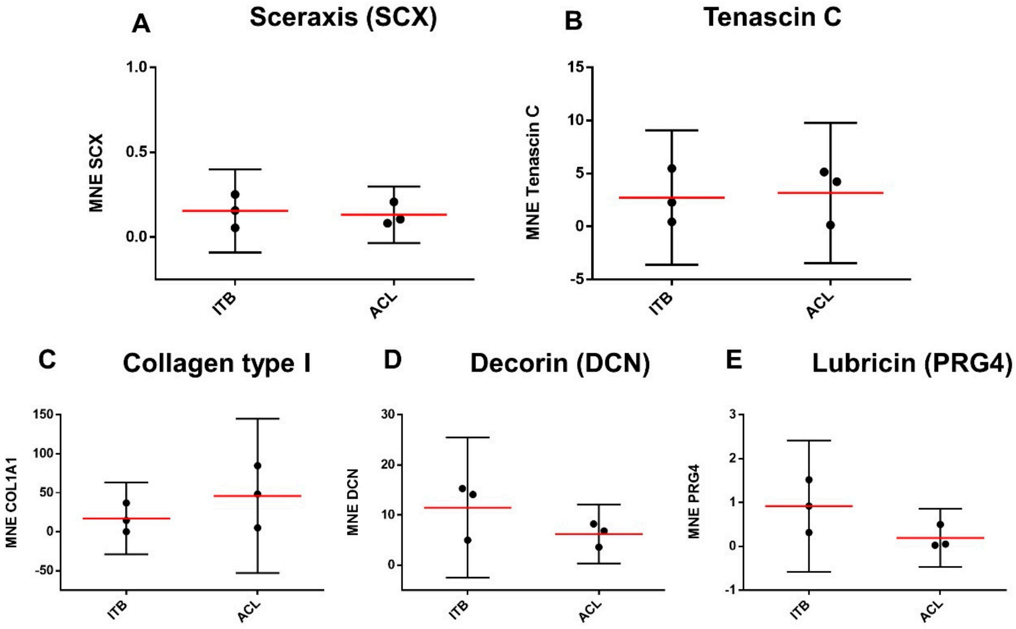

Supplementary Figure 2. Gene expression analyses of the typical ligament components in monolayer-cultured ITB and ACL cells (passages 3–4). The mean normalized expression (MNE) of the tendon marker scleraxis (SCX) (**A**), the fibroblast marker tenascin C (**B**), the ECM proteins collagen type I (COL1A1: collagen type 1 alpha 1 chain) (**C**), decorin (DCN) (**D**), and lubricin (PRG4) (**E**) was determined by using RTD PCR. Hypoxanthine-guanine phosphoribosyltransferase (HPRT) served as a reference gene.
